# Metabolite Profiling: A Tool for the Biochemical Characterisation of *Mycobacterium* sp.

**DOI:** 10.3390/microorganisms7050148

**Published:** 2019-05-25

**Authors:** Margit Drapal, Paul D. Fraser

**Affiliations:** School of Biological Sciences, Royal Holloway University of London, Egham Hill, Egham TW20 0EX, UK; Margit.Drapal.2011@live.rhul.ac.uk

**Keywords:** mycobacteria, metabolomics, anti-TB drugs

## Abstract

Over the last decades, the prevalence of drug-resistance in *Mycobacterium tuberculosis* (*Mtb*), the causative agent of tuberculosis, has increased. These findings have rekindled interest in elucidating the unique adaptive molecular and biochemistry physiology of *Mycobacterium*. The use of metabolite profiling independently or in combination with other levels of “-omic” analyses has proven an effective approach to elucidate key physiological/biochemical mechanisms associated with *Mtb* throughout infection. The following review discusses the use of metabolite profiling in the study of tuberculosis, future approaches, and the technical and logistical limitations of the methodology.

## 1. Introduction

**Figure 1:**
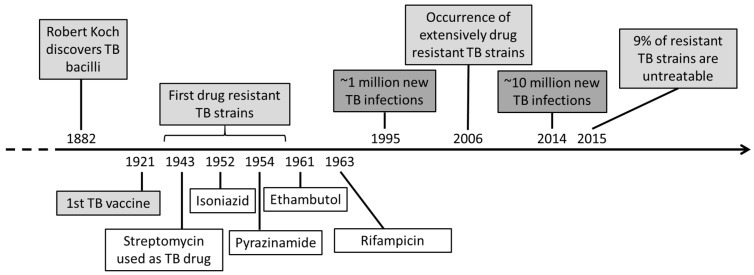
Timeline of tuberculosis (TB) from its discovery to treatments, current case numbers and TB research.

*Mycobacterium tuberculosis* (Mtb) is the causative agent for tuberculosis (TB), causing millions of new infection cases and deaths every year [1]. Since its discovery in 1882 by Robert Koch, many studies have focused on the understanding of the bacilli as well as the treatment and prevention of TB infection (Figure 1). In 1921, the first TB vaccine (*Mycobacterium bovis* Bacillus Calmette-Guérin) was discovered, followed by the first anti-TB drugs during WWII [2,3]. Nevertheless, almost a century later, research is still focused on the eradication of the TB epidemic.

The unique properties of pathogenic mycobacteria provide an advantage over the progression of the infection and against drugs [4,5]. The most prominent properties are the nonreplicating state (NRP), during which the bacilli reduce their metabolic activity to enable long-term viability [6,7], and the mycobacterial cell envelope, which undergoes structural and functional changes under oxygen limiting conditions [8]. The lipid layers of the cell wall form a considerable barrier for the transport of compounds into the cell, preventing drugs from reaching their intracellular targets [7,9,10]. Additionally, the number of mycobacteria developing multidrug-resistance (MDR) to the standard anti-TB drugs increased rapidly over the last few decades [11]. The cause of resistance is known for most of these standard drugs and has resulted in renewed interest for alternative drug target sites [12]. Hence, an important component of studies for new TB therapeutics is the detailed understanding of the metabolism of bacilli across their life cycle [13].

## 2. Metabolite Profiling a “New” Approach for Drug Discovery

**Figure 2:**
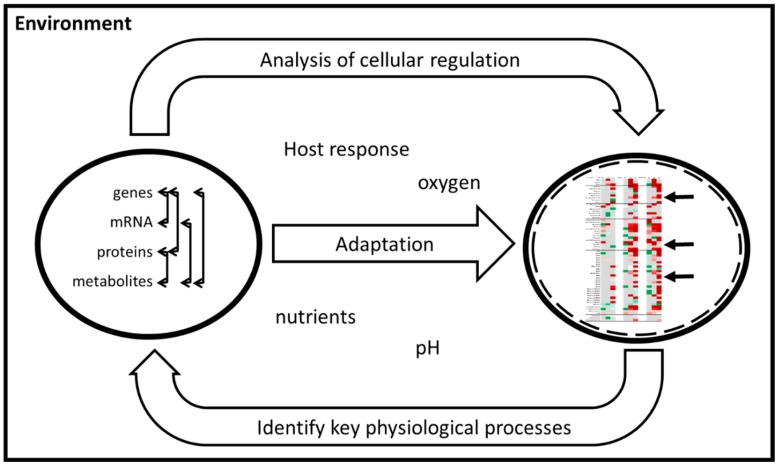
Role of systems biology in understanding key physiological processes of the TB bacilli and intracellular regulation under adaptation to the environment. Arrows represent interaction of intracellular regulation molecules (left circle) and changes of metabolites (right circle).

Bacteria are unicellular systems but still have complex cellular regulatory networks that require analysis at different levels (genome, transcriptome, proteome and metabolome) in order to gain a more holistic understanding of the processes involved. Systems Biology as a discipline has evolved and aims to decipher relationships between the different facets of cellular regulation. Underpinning knowledge, from the understanding of the dynamic behaviour of the system as a whole and interactions between the cell/pathogen and its environment/host (Figure 2), can be exploited in the design of new antibiotics [14,15,16]. Many studies, such as the *Mtb* genome scale model (GSMN), have highlighted that metabolic analysis is needed for a comprehensive analysis and to fill gaps in the reactions predicted from genome annotation [17,18,19].

The metabolome comprises small molecular weight molecules (e.g., sugars) as well as components of larger macromolecules (e.g., amino acids for proteins). Metabolic analysis represents a measure of these compounds and components involved in cellular regulation [20] and can be divided into three different types: Chemical fingerprinting (general screen of the metabolome), metabolite profiling (detailed analysis of a defined group of metabolites) and targeted analysis (accurate analysis of specific metabolites) [21]. The analytical platforms for all metabolic analysis include chromatography often coupled to mass spectrometry. To minimise the analytical procedures, the platform applied needs to be able to analyse metabolites varying in mass and polarity. The more advanced the methods become, the easier it is to compare detected features to published metabolite libraries [22,23,24]. Additionally, the aim of the study defines on which metabolite class the analytical focus is based (e.g., end-products such as lipids or metabolites associated with intermediary metabolism) and contributes to the analytical platform used or utilised [22,25,26].

### 2.1. Understanding Mtb Properties through Metabolite Studies

**Figure 3:**
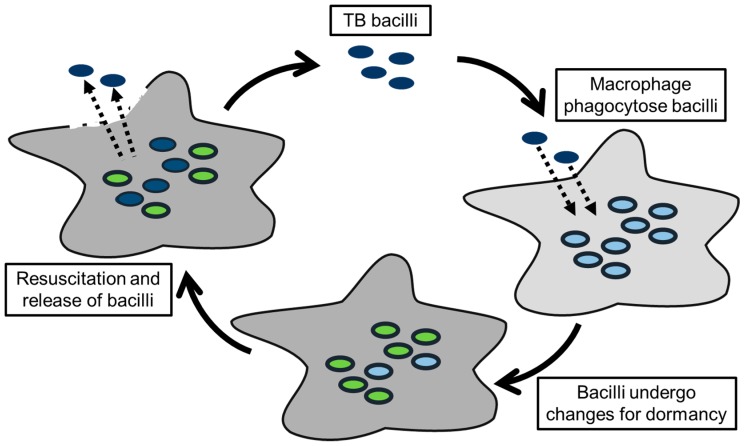
TB infection cycle indicates intracellular changes of the bacilli during the different stages of the infection. The first adaptations occur during phagocytosis of the bacilli by the macrophages. The inner environment of the macrophages starts changing, which causes further adaptations of the bacilli and can include the switch to dormant bacilli. The last adaptation includes the resuscitation from dormant to active bacilli and the changes during airborne transmission.

The understanding of pathogens comprises the identification of compounds involved in virulence as well as the elucidation of intracellular changes throughout the infection cycle (Figure 3). The main compounds related to virulence in *Mtb* are associated with the cell wall and its remodelling/stabilisation during the infection of macrophages. The thickening of the cell wall (higher cross-linking of the peptidoglycan) and modification of cell wall lipids promotes the cell wall rigidity and enables survival in the hostile granuloma environment with a low oxygen content and an acidic pH [27,28]. Many of these lipids (e.g., sulfolipids and trehalose dimycolates) act as virulence factors and induce an immune response in the infected tissue [22,29]. Metabolic analysis facilitated discovery of other cell wall components directly and indirectly involved in virulence. These components are isopentenyl pyrophosphate (IPP)-derived compounds, such as polyprenol carrier lipids, menaquinone, isotuberculosinol and carotenoids, and are involved in the transport of saccharides and mycolic acids for the cell wall, electron transport chain, arrest of phagosomal maturation as well as free radical scavenging [30,31,32,33]. A reduction or even lack of these compounds has been reported to lead to defects of the lipoglycan biosynthesis and a reduction of virulence [34,35,36,37]. In mycobacteria, IPP is produced via a different pathway to that found in human cells, which presents a valuable exploitable drug target [38].

**Figure 4:**
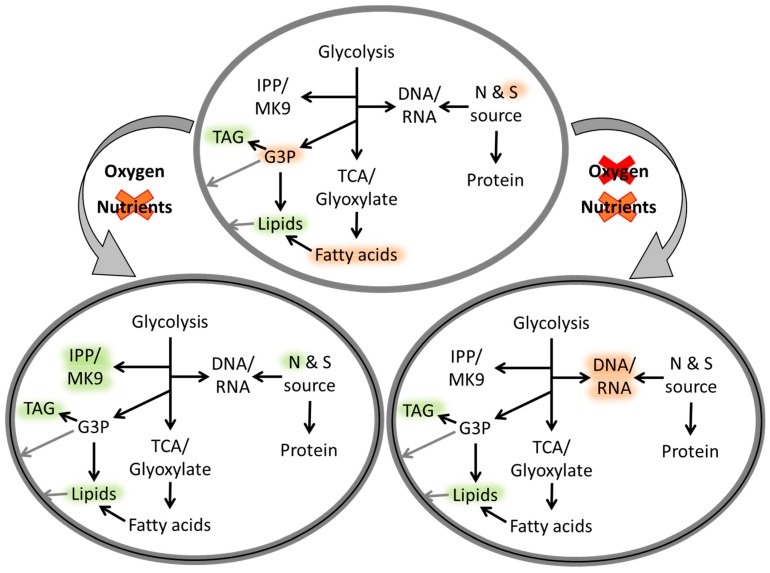
Schematic representation of metabolic processes of mycobacterial cells under changes/depletion of oxygen and nutrients levels (grey curved arrows), adapted from Drapal, M.; et al. *Microbiology* **2016**, *162*, 1456–1467. Copyright © 2016 Microbiology Society [45]. Intracellular metabolic processes are indicated with black arrows and regulation highlighted as up (**green**) or down (**red**).

Over the course of the infection, a significant reprogramming of transcription in *Mtb* can be detected and is primarily influenced by the availability of nutrients and oxygen at each infection stage [39]. The resulting intracellular changes at these stages included the reduction of metabolic activity/transcription, the redirection of the carbon flow and the induction of alternative metabolic pathways, such as the glyoxylate shunt and methyl citrate cycle [7] (Figure 4). The purpose of this reprogramming was attributed to the utilisation of alternative carbon sources (e.g., lipids from the host) and eliminated by-products (e.g., propanoate created during the β-oxidation of odd-number chain fatty acids and production of triacylglycerides as carbon/energy storage) [40,41]. One key enzyme related to most of the processes mentioned was identified as isocitrate lyase (ICL) that converts isocitrate and methyl isocitrate for the alternative TCA cycles [42]. ICL-deficient mycobacteria accumulate precursors associated with these pathways, which leads to bactericidal enzyme inhibition [43].

The culture conditions mimicked by many studies involve a partially aerated or hypoxic NRP state, typically designated on the basis of protocols from Wayne and Hayes [44], which documented an orderly downregulation of the intermediary metabolism, e.g., [27]. Other studies comparing changes of metabolite levels reported a transcriptional mode of regulation under aerated conditions and a more complex metabolic regulation in the presence of hypoxic conditions, e.g., [45]. Despite evident discrepancies about the mode of regulation in previous studies, overall data identified compounds utilised as storage compounds (e.g., succinic acid, triacylglycerides and poly glutamic acid) and arising during the immediate response to resuscitation. Most of these compounds accumulate intracellularly, but some are transported outside the cell, to reduce toxicity and resulting cellular dysfunction [45,46,47]. The process of resuscitation, which can occur quite suddenly [39], is still poorly understood, as it is difficult to establish the exogenous factors influencing this process [7].

### 2.2. New Drug Targets and Understanding Drug Resistance Based on Metabolic Studies

Much progress has been achieved in identifying new anti-TB drugs and drug targets. However, despite the promising results, many drugs against MDR *Mtb* strains (e.g., d-cycloserine) tend to lead to serious side effects in the host [48]. In general, research for anti-TB drugs has been focused on intracellular targets both under aerobic and hypoxic conditions and on weakening the mycobacterial cell wall for more drug accessibility. This led to the discovery of metronidazole, which targets DNA in anaerobe bacteria without antagonistic effects in combination with isoniazid or rifampicin [49]. Other studies identified methionine synthesis, important for nucleotide and protein synthesis, as a viable target, as human cells do not synthesize this amino acid [50,51]. The permeability of the cell wall can be influenced by a diverse range of compounds. Examples are trehalose, a saccharide utilised as backbone for mycolic acids, and IPP-derived carrier lipids for cell wall components. The latter can be affected by the inhibition of IPP synthesis through fosmidomycin, a known malaria drug [52,53]. In addition to the permeability, efflux pumps play an important role in decreasing the concentration and therefore efficiency of drugs already within the bacterial cell [54]. Metabolite profiling offers a means of assessing known inhibitor targets, secondary targets and the holistic effects of these drugs/inhibitors on the metabolome under different conditions. In combination with standardised metabolite profiles for known well-characterised drugs, metabolomics can also be used to classify new chemical inhibitors and elucidate their modes of action [55]. Additionally, the metabolic analysis of resistant mycobacteria can help to understand why the resistance occurs and whether/how it can be circumvented as reviewed previously [56,57,58].

## 3. Advantages and Disadvantages of Metabolite Profiling Studies

### 3.1. Model Organisms for Mtb

*Mtb* is logistically difficult to work with due to biosafety risks and slow growth. Hence, suitability of model organisms has led to intense debates [59,60,61]. The most common model species are *Mycobacterium smegmatis* and *M. bovis* BCG. *M. smegmatis* is a fast-growing species and has ~1.7-times larger genome than *Mtb* [62,63]. *M. smegmatis* is nonpathogenic, even though it shares 12 out of 19 virulence gene homologues found in *Mtb.* This challenges the adequacy of *M. smegmatis* for pathogenicity studies [61]. Furthermore, *M. smegmatis* develops a spore-like shape under oxygen limitation with a cell morphology different to slow-growing mycobacteria [64]. *M. bovis* BCG is a slow-grower and closely related to *Mtb.* They share ~99.9% of the DNA and, for unknown reasons, *M. bovis* BCG never regained virulence [4,65]. A comparison of five *Mycobacterium* species (*M. bovis* BCG, *Mycobacterium avium*, *Mycobacterium intracellulare*, *M. smegmatis* and *Mycobacterium phlei*) with different growth rates and pathogenicity, showed that: (i) Their only common trait was the metabolic processes related to the cell wall and (ii) the metabolic changes under aerobic and hypoxic conditions emphasise differences on a genetic level between fast- and slow-growing mycobacteria [45]. Conclusions from other studies highlighted that the model *Mycobacterium* species for *Mtb* depends on the purpose of the metabolic study, and the use of phenotypically closer-related species (e.g., *M. bovis* BCG) is recommended for specialised pathways. Nevertheless, a metabolic comparison of nonpathogenic and pathogenic mycobacteria might provide valuable insights into virulence and ‘dormancy’, as the processes involved (e.g., glyoxylate shunt, central carbon metabolism) often also occur in nonpathogenic mycobacteria [42,59,66]. Metabolomic studies facilitate chemotyping different/diverse strains and identifying subtle differences in their steady-state metabolite levels that can eventually be utilised as differentiating biomarkers [42].

### 3.2. Heterogeneous Cultures

The phenomenon of heterogeneous mycobacterial cultures, comprising active and ‘dormant’ bacilli, bears an obstacle for data interpretation of metabolite profiling [67]. The detected metabolite changes of the culture/tissue samples contain information for both states which cannot be related to one single state and, hence, does not give accurate details about the processes involved in transition between active and ‘dormant’ bacilli. Previous studies implied ‘dormancy’ as a redirection of metabolic activity under unfavourable growth conditions, e.g., [46]. The renewed growth rate and metabolic activity, detected after 28 days under aerated and hypoxic conditions, indicate that the process of ‘dormancy’ does not involve the whole culture [45]. Further analysis of such growth conditions is of importance for a better understanding of the resuscitation process, which could just reside from a delayed log phase of very few active bacilli in the culture instead of the assumed complex and sudden occurrence [7,39].

### 3.3. In Vitro Versus in Vivo Studies

Metabolite profiling can not only analyse cellular compounds but also the culture composition affecting the metabolism of the strains. In vitro studies represent a regulated environment with set parameters mimicking an in vivo study, which includes a far broader range of parameters and influencing factors, of which many can be potentially unknown. In the case of a *Mtb* infection, these parameters include all the stages experienced from active replication in the alveolar macrophages to dormant cells within granulomas [7]. Hence, an in vitro study mimicking set conditions might be more helpful, as the first step to elucidate complex processes (e.g., ‘dormant state’ of mycobacteria) compared to an in vivo study. A main disadvantage of an in vitro study is cell concentration, which is usually higher in in vitro studies compared to human sputum with 10^1^–10^5^ cells/ml sputum [68,69].

The introduction of metabolomics for TB research led to major advances in diagnostic tools and the continuous replacement of tradition methods such as sputum smear tests with non-invasive techniques [70,71]. The increasing number of metabolic studies led to the discovery of new TB biomarkers (e.g., volatiles and specific lipids) in plasma and urine samples [24,72]. The advancement of high-resolution metabolomics can distinguish between human and TB metabolites, elucidating the progression of TB in the human host and the host response [73]. Furthermore, the higher resolution of the technique facilitates the identification of biomarkers to a high confidence level with the libraries available [74].

### 3.4. Limitation to Metabolite Profiling

The metabolome comprises a broad range of compounds that differ in their properties and dynamic range. A complete study of the whole metabolome would require analysis with different platforms and techniques such as steady-state levels or fluxomics [23,26,75]. Studies focused on an overview of the metabolic steady-state levels have shown a strong correlation to the literature and endorsed the importance of metabolic studies [45]. Hence, it is of great value to integrate recently acquired results with published data concerning metabolic biology studies as well as those representing other levels of cellular regulation. This should also include techniques such as electron microscopy to gain a comprehensive understanding of cell biology and how it is influenced by changes in metabolite composition [6].

## 4. Conclusions

The consistent threat of the TB epidemic and the occurrence of resistance to existing antibiotics challenges the community to better understand *Mtb* and its infection cycle. Metabolite profiling is a new tool in this research area that can be used to elucidate key physiological properties of *Mtb* throughout infection. To date, the most prevalent studies relate to cell wall metabolism and the production of storage compounds during ‘dormancy’. The metabolomics platforms now established can be used to (i) generate characteristic biochemical signatures for specific *Mycobacterium* strains, (ii) identify specific developmental stages in their life cycle and (iii) elucidate metabolic adaptation to their environment. Despite these notable advances, the need for better metabolite annotation and integration with other “omic” technologies to generate exploitable network models remains.
